# Does CHA_2_DS_2_-VASc Score Predict MACE in Patients Undergoing Isolated Coronary Artery Bypass Grafting Surgery?

**DOI:** 10.21470/1678-9741-2018-0323

**Published:** 2019

**Authors:** Muhsin Kalyoncuoglu, Semi Ozturk, Mazlum Sahin

**Affiliations:** 1 Department of Cardiology, Haseki Training and Research Hospital, Istanbul, Turkey.; 2 Department of Cardiovascular Surgery, Haseki Training and Research Hospital, Istanbul, Turkey.

**Keywords:** Coronary Artery Disease, Coronary Artery Bypass – adverse effects, Lenght of Stay – trends, Time Factors

## Abstract

**Objective:**

To evaluate the prognostic value of CHA_2_DS_2_-VASc score in individuals undergoing isolated coronary artery bypass grafting (CABG) surgery.

**Methods:**

Records of consecutive 464 patients who underwent elective isolated CABG, between January 2015 and August 2017, were retrospectively reviewed. A major adverse cardiac event (MACE) was the primary outcome of this study. MACE in patients with low (L) (<2, n: 238) and high (H) (≤2, n: 226) CHA_2_DS_2_-VASc scores were compared. Univariate logistic regression analysis identified preditors of MACE.

**Results:**

Hypertension, diabetes mellitus, and peripheral vascular disease were more frequent in the H group than in the L group. European System for Cardiac Operative Risk Evaluation (EuroSCORE) I and SYNTAX I scores were similar in both groups while SYNTAX II-CABG score was significantly higher in the H group than in the L group. Postoperative myocardial infarction, need for intra-aortic balloon pump, acute renal failure, and mediastinitis were more frequent in the H group than in the L group. The H group had significantly higher in-hospital mortality and MACE rates than the L group (*P*<0.01). EuroSCORE I, SYNTAX II-CABG, and CHA_2_DS_2_-VASc scores were predictors for MACE. SYNTAX II-CABG > 25.1 had 68.4% sensitivity and 52.7% specificity (area under the curve [AUC]: 0.653, *P*=0.04, 95% confidence interval [CI]: 0.607-0.696) and CHA_2_DS_2_-VASc > 2 had 52.6% sensitivity and 84.1% specificity (AUC: 0.752, *P*<0.01, 95% CI: 0.710-0.790) to predict MACE. Pairwise comparison of receiver-operating characteristic curves revealed similar accuracy for both scoring systems.

**Conclusion:**

CHA_2_DS_2_-VASc score may predict MACE in patients undergoing isolated CABG.

**Table:** 

Abbreviations, acronyms & symbols				
ACS	= Acute coronary syndrome		ICU	= Intensive care unit
AF	= Atrial fibrillation		IQR	= Interquartile range
AUC	= Area under the curve		L	= Low
BMI	= Body mass index		LDL-C	= Low-density lipoprotein cholesterol
CABG	= Coronary artery bypass grafting		LVEF	= Left ventricular ejection fraction
CAD	= Coronary artery disease		MACE	= Major adverse cardiac event
CI	= Confidence interval		MI	= Myocardial infarction
COPD	= Chronic obstructive pulmonary disease		OR	= Odds ratio
CPB	= Cardiopulmonary bypass		PAD	= Peripheral artery disease
CRP	= C-reactive protein		PCI	= Percutaneous coronary intervention
DM	= Diabetes mellitus		PVD	= Peripheral vascular disease
EuroSCORE	= European System for Cardiac Operative Risk Evaluation		ROC	= Receiver-operating characteristic
H	= High		SD	= Standard deviation
HDL-C	= High-density lipoprotein cholesterol		SE	= Standard error
Ht	= Hypertension		SPSS	= Statistical Package for the Social Sciences
HT	= Heart transplantation		STS	= Society of Thoracic Surgeons
IAB	= Intra-aortic balloon		TAVR	= Transcatheter aortic valve replacement

## INTRODUCTION

Coronary artery bypass grafting (CABG) surgery and percutaneous coronary intervention (PCI) are widely used revascularization strategies for coronary artery disease (CAD) which reduce mortality and improve quality of life^[1]^. Recent data suggested the superiority of CABG in preventing major cardiac events in patients with multivessel disease, particularly in patients with complex CAD and diabetes^[2]^.

The European System for Cardiac Operative Risk Evaluation (EuroSCORE) and the Society of Thoracic Surgeons (STS) 2008 Cardiac Surgery Risk Model are the most commonly used risk prediction models for cardiac surgery. These scoring systems are not only useful to assess the effect of specific clinical parameters on surgical outcomes, but also to aid in treatment selection, patient counseling, comparison of postoperative results, and quality improvement^[3]^. Besides, current mortality risk prediction models for CABG do not have a standardized approach in terms of both defining predictor variables and outcome. In addition, some problematic topics such as inadequate sample size, inappropriate handling of missing data, as well as suboptimal statistical techniques make these risk models debatable^[4]^. Need for calculator or computer make these models impractical for daily clinical use. Therefore, more practical risk modeling systems which predict morbidity and mortality are required.

CHADS_2_ and CHA_2_DS_2_-VASc scores are well-validated and proposed scoring systems to establish the risk of stroke in patients with non-valvular atrial fibrillation (AF). Additionally, CHA_2_DS_2_-VASc score components, such as age, hypertension, diabetes mellitus (DM), and prior cardiovascular event, are also traditional risk factors for CAD. Previous studies demonstrated the association between the CHA_2_DS_2_-VASc score and the severity of CAD^[5]^. Recent studies demonstrated the prognostic value of the CHA_2_DS_2_-VASc score in patients suffering from acute coronary syndrome (ACS)^[6]^.

Although CHA_2_DS_2_-VASc score is proposed as a predictor for immediate and late stroke after CABG, there is no data evaluating the prognostic value of CHA_2_DS_2_-VASc score in patients undergoing isolated CABG surgery^[7]^. When compared with the aforementioned risk models, the CHA_2_DS_2_-VASc score is a fast and simple method for risk evaluation that requires no calculator or computers. We sought for the prognostic value of CHA_2_DS_2_-VASc score in individuals undergoing isolated CABG surgery.

## METHODS

### Study Population

This study included patients who underwent isolated CABG at the Haseki Training and Research Hospital between January 2015 and August 2017. The study excluded patients with concomitant other surgeries such as valve repair or replacement. Patients with preoperative AF were also excluded. Emergent procedures were excluded since preoperative assessments, such as carotid ultrasound, were insufficient. Records of 555 patients were retrospectively reviewed. Of these, 22 (3.9%) patients had an insufficient record, 14 (2.5%) underwent off-pump surgery, and 23 (4.1%) underwent concomitant other cardiac surgery (valvular, ventricular aneurysms, acquired ventricular septal defect). Additional 32 (5.8%) patients who underwent emergent surgery were excluded from the study. All patients were operated by the same group of cardiovascular surgeons and anesthesiologists. Same techniques during CABG and myocardial protection were used.

The study population was retrospectively and consecutively analyzed by using our database, which was collected as a part of routine clinical practice. Data from each patient were obtained from a computerized system or a patient file. Demographic and laboratory variables including age, gender, body mass index (BMI), C-reactive protein, lipid panel, and clinical variables during hospitalization were recorded. Clinical variables included cardiopulmonary bypass (CPB) time, need for intra-aortic balloon (IAB), clamp time, total number of grafts, extubation time, bleeding revision, perioperative myocardial infarction (MI), sternal dehiscence, wound infection, cerebrovascular event (stroke or transient ischemic attack), mediastinitis, acute kidney injury, acute AF (lasting longer than one hour), intensive care unit (ICU) time, hospitalization time, and in-hospital mortality.

### Risk Scores

#### SYNTAX I-II Score

The angiograms of the patients were evaluated by two experienced interventional cardiologists who were blind to the study. CAD was defined as a stenosis of more than 50% of the lumen diameter in any of the main coronary arteries. SYNTAX I-II scores were calculated by using the downloaded version (www.syntaxscore.com).

#### EuroSCORE I

Preoperative risk stratification was performed for all patients by using the downloaded version of the EuroSCORE system (euroscore.org).

#### CHA_2_DS_2_-VASc Score

CHA_2_DS_2_-VASc score was calculated for all patients by assigning one point for each of the following criteria: age 65-75 years, hypertension, DM, congestive heart failure or left ventricular ejection fraction (LVEF) < 40%, female sex, and vascular disease (defined as prior MI, complex aortic plaque, carotid disease, peripheral artery disease including intermittent claudication, and previous surgical or percutaneous intervention for abdominal aorta or vessels of upper or lower extremities). Two points were assigned for a history of stroke or transient ischemic attack or thromboembolism and age ≥ 75 years. Since all patients underwent coronary bypass surgery due to multiple CAD, CAD at index hospitalization was not taken into account. After the CHA_2_DS_2_-VASc score calculation, the study population was divided into two groups: low (L) (CHA_2_DS_2_-VASc <2 ) and high (H) (CHA_2_DS_2_-VASc ≥ 2) score groups.

### Study Endpoints

A major adverse cardiac event (MACE) was the primary endpoint of this study. MACE was defined as a composite of in-hospital mortality, postoperative non-fatal MI, cardiac arrest requiring cardiopulmonary resuscitation, need for new mechanical circulatory support, and cerebrovascular event during intraoperative/postoperative hospitalization. In-hospital mortality was defined as death from all causes during intraoperative and postoperative hospitalization. The study was approved by the local ethics committee.

### Statistical Analysis

Statistical analyses were performed with Statistical Package for the Social Sciences (SPSS) software version 22.0 (IBM Corp. Armonk, New York, United States of America) and MedCalc bvba version 16 (Seoul, Korea). Normality of the data was analyzed with the Kolmogorov-Smirnov test. Continuous data were expressed as mean ± standard deviation (SD) and categorical data were expressed as percentages. Categorical variables between the groups were assessed with Chi-square test or Fisher’s exact test, whichever was suitable. Logistic regression analysis was used to identify the independent predictors of MACE. Differences between patient subgroups were tested using Mann-Whitney U test or Student's t-test, where appropriate. A *P*-value < 0.05 was considered statistically significant. Receiver-operating characteristic (ROC) curve graphics were used to determine the cut-off values of predictors for MACE.

## RESULTS

Four hundred sixty-four patients who underwent elective isolated CABG surgery were included in the study. Patients were dichotomized depending on their CHA_2_DS_2_-VASc score. L and H score groups were compared as previously described. The L group included 238 patients (median age: 57 years [interquartile range {IQR}: 52-63]; 44 [18.5%] females) while the H group included 226 patients (median age: 64 years [IQR: 55-67]; 45 [19.9%] females). Hypertension, DM, and peripheral vascular disease (PVD) were more frequent in the H group (*P*<0.001, *P*<0.001, *P*=0.044, respectively) than in the L group. EuroSCORE I was similar in both groups (*P*=0.53). Anatomical based SYNTAX I score was similar in both groups, while clinical SYNTAX II-CABG score was significantly higher in the H group than in the L group (*P*=0.4, *P*=0.001, respectively). Postoperative MI was more frequent in the H group (*P*=0.006) than in the L group. Patients in the H group needed more İAB pump support (*P*=0.005) than those in the L group. Acute renal failure and mediastinitis in the postoperative period were more frequent in H group (*P*<0.001, *P*<0.001, respectively) than in the L group. Clinical, laboratory, and operative parameters were presented in Table 1 and Table 2. The H group had significantly higher in-hospital mortality and MACE rates than the L group (*P*<0.01).

**Table 1:** Demographic, clinical, and laboratory characteristics of the groups.

	CHA_2_DS_2_-VASc		*P*
	<2 n=238	≤2 n=226	
Sex (female), n (%)	44 (18.5)	45 (19.9)	0.32
Age (years)	57 (52-63)	64 (55-67)	<0.001
Body mass index (kg/m^2^)	27 (24.3-29.7)	26.4 (24.2-29.5)	0.19
Smoking, n (%)	99 (41.6)	82 (36.3)	0.24
DM, n (%)	19 (8)	143 (63.3)	<0.001
Ht, n (%)	38 (16)	147 (65)	<0.001
COPD, n (%)	34 (14.3)	38 (16.8)	0.45
PAD, n (%)	14 (5.9)	43 (19)	0.044
CAD, n (%)	36 (15.1)	41 (18.1)	0.38
Stroke or transient ischemic attack, n (%)	14 (5.9)	9 (4)	0.35
Ejection fraction (%)	55 (50-60)	45 (40-60)	0.03
Total cholesterol	240 (200-356)	226 (208-310)	0.47
LDL-C	116 (99.5-175)	116 (100-175)	0.94
HDL-C	45 (42-49)	45 (42-51.8)	0.48
CRP	5.5 (4-10)	5 (4-9)	0.70
EuroSCORE I	3 (2-4)	3 (2-4)	0.53
SYNTAX I	19 (14-25.5)	19 (13-23.5)	0.40
SYNTAX II-CABG	23.8 (21.3-28)	25.7 (21.7-35)	0.001

CABG=coronary artery bypass grafting; CAD=coronary artery disease; COPD=chronic obstructive pulmonary disease; CRP=Creactive protein; DM=diabetes mellitus; EuroSCORE=European System for Cardiac Operative Risk Evaluation; HDL-C=high-density lipoprotein cholesterol; Ht=hypertension; LDL-C=low-density lipoprotein cholesterol; PAD=peripheral artery disease

**Table 2:** Operative and postoperative parameters of the groups.

	CHA_2_DS_2_-VASc		*P*
	<2 n=238	≤2 n=226	
Bypass number	3 (2-3)	3 (2-3)	0.36
CPB time (min)	92.5 (47-96)	91 (47-96)	0.16
Clamp time (min)	50 (26-55)	48 (26-56)	0.58
Intra-aortic balloon pump, n (%)	3 (1.3)	14 (6.2)	0.005
Extubation time (hours)	7 (5-9.5)	7 (5-10)	0.63
Bleeding revision, n (%)	9 (3.8)	14 (6.2)	0.23
Hemorrhage (ml)	500 (350-600)	450 (350-600)	0.73
Sternal dehiscence, n (%)	13 (5.5)	10 (4.4)	0.724
Wound ınfection, n (%)	12 (5)	12 (5.3)	0.89
Mediastinitis, n (%)	0 (0)	12 (5.3)	<0.001
Acute renal failure, n (%)	1 (0.4)	14 (6.2)	<0.001
Acute atrial fibrillation, n (%)	27 (11.3)	25 (11.1)	0.92
Transient ischemic attack, n (%)	0 (0)	4 (1.9)	0.056
Stroke, n (%)	1 (0.4)	4 (1.8)	0.34
Post-operative MI, n (%)	0 (0)	7 (3.1)	0.006
In-hospital mortality, n (%)	0 (0)	14 (6.2)	<0.01
MACE, n (%)	1 (0.4)	18 (8)	<0.01
Intensive care unit time (day)	2 (2-2)	2 (2-3)	0.91
Hospitalization time (day)	5 (5-6)	5 (5-6)	0.23

CPB=cardiopulmonary bypass; MACE=major adverse cardiac events; MI=myocardial infarction

When each component of CHA_2_DS_2_-VASc score was analyzed in univariate logistic regression analysis, congestive heart failure or ejection fraction < 40%, age, hypertension, PVD, and cerebrovascular event were independent predictors for MACE (Table 3).

**Table 3:** Logistic regression of each CHA_2_DS_2_-Vasc score component for MACE.

Variables		*P*		OR (95% CI)	
Congestive heart failure or ejection fraction < 40%		<0.001		12.3 (3.37- 44.87)	
Sex		0.87			
Age		0.01		1.13 (1.03-1.24)	
Hypertension		0.008		5.68 (1.56-20.61)	
Diabetes mellitus		0.08			
Cerebrovascular event (stroke or transient ischemic attack)		<0.001		11.42 (3.5-37.28)	
Peripheral vascular disease		0.01		4.37 (1.4-1.52)	

CI=confidence interval; OR= *odds ratio*

**Fig. 1:**
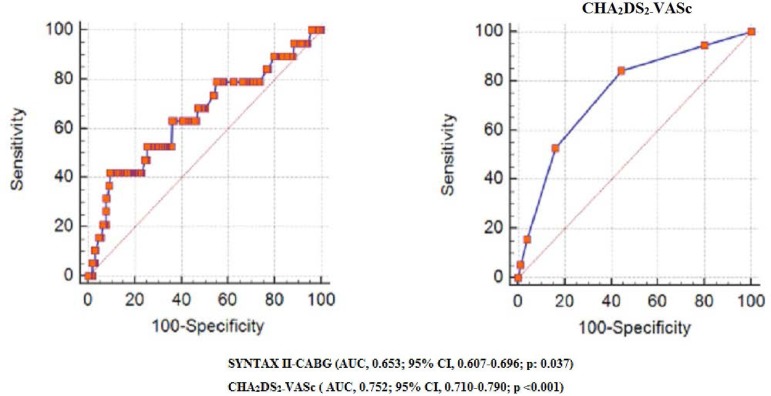
Receiver-operating characteristic curve analysis of SYNTAX II-CABG and CHA_2_DS_2_-VASc for prediction of a major adverse cardiac event. AUC=area under the curve; CABG=coronary artery bypass grafting; CI=confidence interval

**Fig. 2:**
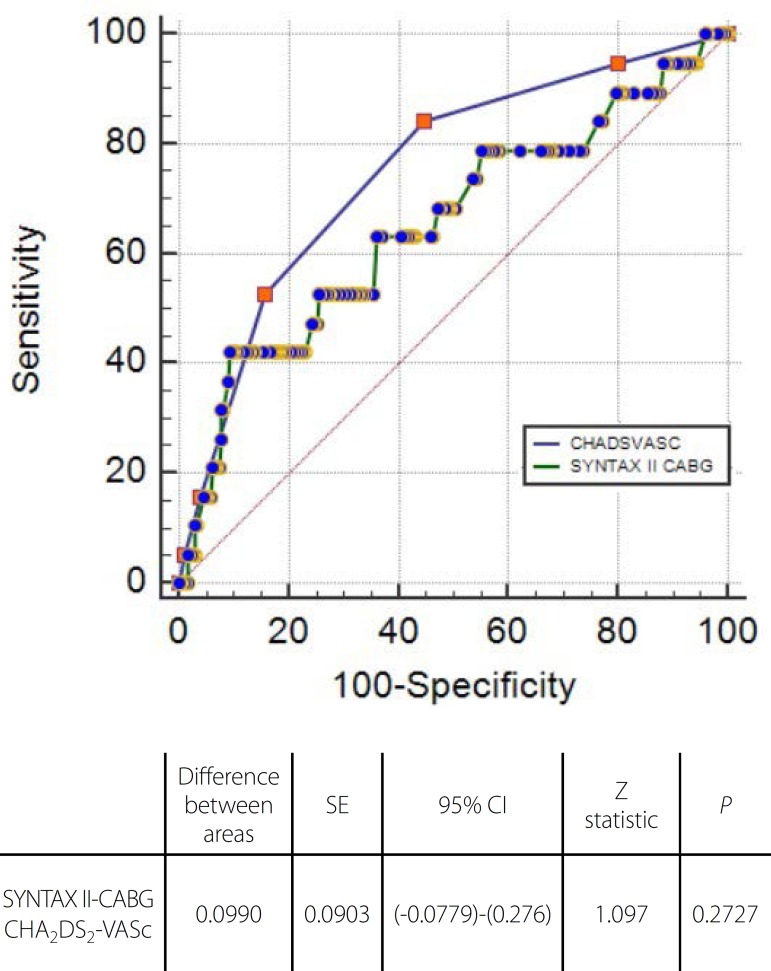
Pairwise comparison of receiver-operating characteristic curves of SYNTAX II-CABG and CHA^2^DS_2_-VASc for prediction of a major adverse cardiac event. CABG=coronary artery bypass grafting; CI=confidence interval; SE=Standard error

We performed univariate analysis including EuroSCORE I, SYNTAX I and SYNTAX II, and CHA_2_DS_2_-VASc scores. EuroSCORE, SYNTAX II-CABG, and CHA_2_DS_2_-VASc scores were the predictors for MACE in logistic regression analysis (Hosmer-Lemeshow test, *P*=0.414, 0.941, and 0.693, and Nagelkerke R Square, *P*=0.359, 0.047, 0.105; respectively) (Table 4). ROC curve analysis of SYNTAX II-CABG and CHA_2_DS_2_-VASc scores were performed to predict MACE (Figure 1). SYNTAX II-CABG > 25.1 had 68.4% sensitivity and 52.7% specificity to predict MACE (area under the curve [AUC]: 0.653, *P*=0.04, 95% confidence interval [CI]: 0.607-0.696). CHA_2_DS_2_-VASc > 2 had 52.6% sensitivity and 84.1% specificity to predict MACE (AUC: 0.752, *P*<0.01, 95% CI: 0.710-0.790) (Table 5). We compared CHA_2_DS_2_-VASc with SYNTAX II-CABG, which is a relatively and comprehensive score. Pairwise comparison of ROC curves revealed the similar statistical accuracy of both scoring systems for prediction of MACE (Z statistic: 1.097, *P*=0.27) (Figure 2).

**Table 4:** Predictors of major adverse cardiac events; univariate logistic regression analysis of CHA_2_DS_2_-VASc, SYNTAX I, and SYNTAX II scores.

Variables	Univariate analysis OR (95% CI)	*P * value
SYNTAX I	1.00 (0.98-1.03)	0.83
SYNTAX II-PCI	1.01 (0.99-1.02)	0.30
SYNTAX II-CABG	1.07 (1.02-1.12)	0.01
EuroSCORE I	2.71 (1.95-3.75)	<0.001
CHA_2_DS_2_-VASc	2.03 (1.41-2.92)	<0.001

CABG=coronary artery bypass grafting; CI=confidence interval; EuroSCORE=European System for Cardiac Operative Risk Evaluation; OR= *odds ratio* ; PCI=percutaneous coronary intervention

**Table 5:** Sensitivity and specifity of CHA_2_DS_2_-VASc to predict MACE.

Criterion	Sensitivity	95% CI	Specificity
≥0	100	82.4-100	0.00
>0	94.74	74.0-99.9	19.96
>1	84.21	60.4-96.6	55.38
>2	52.63	28.9-75.6	84.08
>3	15.79	3.4-39.6	95.96
>4	5.26	0.1-26.0	98.88
>5	0.00	0.0-17.6	99.78
>6	0.00	0.0-17.6	100

CI=confidence interval; MACE=major adverse cardiac event

## DISCUSSION

We demonstrated, for the first time, that CHA_2_DS_2_-VASc score is an independent predictor for MACE in patients undergoing isolated CABG. Although CHA_2_DS_2_-VASc score includes only clinical parameters, it is as accurate as SYNTAX II-CABG, which includes a detailed angiographic evaluation.

CABG is a safe procedure with low rates of mortality and morbidity. However, the ability to accurately predict adverse outcomes and short- and long-term mortalities after CABG is an important issue that may allow planning preventive strategies and minimize complications^[8]^. EuroSCORE, STS risk calculator, and Parsonnet score are the most commonly used risk stratification models which include multiple variables, requiring online calculators for estimation of risk-related mortality and morbidity with CABG^[9]^. Nevertheless, these are complex and impractical tools to use at the bedside. Therefore, we still need models to quickly and easily predict risk at bedside, without the need for computational software. In practice, the bedside risk assessment not only provides the surgeon an objective, measurable risk profile to identify patients who require meticulous care, but it also provides the patients a more detailed knowledge of the risk related with the surgical procedure^[10]^.

CHA_2_DS_2_-VASc score is an easily applicable time-saving risk model which predicts the risk of thromboembolic events in patients with non-valvular AF in daily practice. CHA_2_DS_2_-VASc score components, such as older age, female gender, hypertension, DM, extracardiac arteriopathy, low LVEF, and preoperative stroke, and the presence of CAD were reported as predictors of early outcomes after CABG^[11]^.

The female sex has been reported as an independent predictor of short- and long-term mortalities and adverse events after CABG^[12]^. Although the female sex is included in both STS and EuroSCORE, evidence is controversial. Several studies concluded that the female sex is not a risk factor for post procedural mortality after CABG^[13]^. In our study, the female sex was not found to be associated with MACE, which is compatible with previous studies.

Increased age (> 60 years) has been reported as an independent predictor of mortality and adverse events after CABG^[14]^. In the EuroSCORE model, 60 years of age was accepted as a cut-off value, thus one point was assigned per each five years above 60 years. In the CHA_2_DS_2_-VASc score, one point was assigned for 65-75 of age years and two points were assigned for age ≥ 75 years. Compatible with previous studies, our study demonstrated an association between age and MACE^[14]^.

Heart transplantation (HT) and DM are not included in the EuroSCORE risk model. On the other hand, HT, DM, and age were the predominant variables causing high scores in our study. Age and HT were independent predictors for MACE in the present study. Although several studies revealed that patients with DM are at high risk for MACE and death after CABG, univariate analysis revealed that DM was not an independent predictor for MACE in the present study, like studies by Rajakaruna et al.^[15]^. These conflicting data may be related to defining the criteria of DM. DM was defined as the need for insulin or oral medication in the present study, while another study defined DM as the need for diet, oral medication, or insulin therapy^[16]^. Patients with insulin-dependent DM have a significantly higher rate of mortality and MACE than those with non-insulin-dependent DM^[17]^. We also did not classify DM as insulin-dependent or non-dependent. These might explain the different outcome associated with diabetes on early mortality in the present study.

Although there is conflicting data in the literature, we showed that preexisting HT is a poor predictor for patients undergoing isolated CABG^[18]^. CABG has been shown to be superior to medical therapy alone in patients with preoperative low LVEF^[19]^. Besides, Dalen et al. showed that the reduced ejection fraction doubled the risk of early postoperative death^[20]^. Compatible with the previous studies, patients with left ventricular systolic dysfunction and symptoms of heart failure had significantly high in-hospital mortality and morbidity in our study^[9]^.

Similar to our study, numerous studies have demonstrated that the PVD was an independent predictor of early mortality and poor short-term outcome after CABG^[21]^. On the other hand, the association between PVD and early mortality was not confirmed in some studies^[22]^.

History of stroke has been found to be associated with mortality and increased early and late postoperative stroke^[23]^. In line with the literature, the present study demonstrated that a history of preoperative stroke was associated with poor outcome.

Consequently, when we performed univariate analysis of each CHA_2_DS_2_-VASc score component, congestive heart failure or ejection fraction < 40%, age, hypertension, cerebrovascular accident, and PVD were significantly associated with MACE in patients undergoing after isolated CABG, whereas DM and sex were not.

Several studies have demonstrated an association between the CHA_2_DS_2_-VASc score and increased mortality and non-fatal adverse cardiovascular outcomes in different clinical conditions, regardless of the presence of AF^[24]^. Recently, Hamid et al. observed high mortality in the patients undergoing transcatheter aortic valve replacement who had CHA_2_DS_2_-VASc score > 6. Therefore CHA_2_DS_2_-VASc score was proposed as a simple tool for the identification of high-risk patients for short-term and mid-term mortalities in patients undergoing transcatheter aortic valve replacement (TAVR)^[25]^.

This the first study which aimed to investigate the value of CHA_2_DS_2_-VASc score to estimate MACE in patients undergoing isolated CABG surgery. Biancari et al. demonstrated predictive accuracy of CHA_2_DS_2_-VASc score for immediate and late stroke in patients after CABG without pre- and postoperative AF. They also demonstrated that CHA_2_DS_2_-VASc score is a predictor for late all-cause mortality and cardiovascular mortality^[8]^. But there is no data specifically evaluating the value of CHA_2_DS_2_-VASc score for prediction of in-hospital mortality and MACE in patients undergoing isolated CABG.

### Limitation

The major limitation of our study is that we focused on the CHA_2_DS_2_-VASc score, which only includes the preoperative variables, and we did not take operative and postoperative variables into account. In the recent years, more complex preoperative patient profile was being referred for CABG due to improved medical treatment options and achievements of interventional cardiology. As preoperative variables may have limited predictor role without combination with operative and postoperative variables, larger studies including preoperative, operative, and postoperative variables are required. The second limitation was that the surgery was performed by a particular surgeon group. And another limitation is the retrospective nature of the study. So this study is single-centered and may not be generalized to all patient groups.

## CONCLUSION

We showed that CHA_2_DS_2_-VASc score may predict in-hospital mortality and MACE in patients undergoing isolated CABG in our study population. CHA_2_DS_2_-VASc score is a handy risk stratification score and easily appliable at bedside without any computational software. Further studies are required to assess the validity of our findings in larger populations.

**Table:** 

Authors’ roles & responsibilities	
MK	Substantial contributions to the conception or design of the work; or the acquisition, analysis, or interpretation of data for the work; final approval of the version to be published.
SO	Substantial contributions to the conception or design of the work; or the acquisition, analysis, or interpretation of data for the work; final approval of the version to be published.
MZ	Substantial contributions to the conception or design of the work; or the acquisition, analysis, or interpretation of data for the work; final approval of the version to be published.
